# Hepatitis B-related acute-on-chronic liver failure induced by hepatotropic viral insult is associated with worse prognosis than that induced by non-virus insult

**DOI:** 10.1186/s12879-021-06974-z

**Published:** 2021-12-20

**Authors:** Jing Liang, Lei Liu, Yingying Cao, Qian Zhang, Fang Liu, Yu Chen, Hua Liu, Zhongping Duan, Manman Xu, Shaojie Xin, Shaoli You, Fangjiao Song, Jun Li, Tao Han

**Affiliations:** 1 Department of Hepatology, The Third Central Clinical College of Tianjin Medical University, 83, Jintang Road, Tianjin, 300170 China; 2 Department of Hepatology, The Third Central Hospital of Tianjin, Tianjin, China; 3 Tianjin Key Laboratory of Extracorporeal Life Support for Critical Diseases, Tianjin, China; 4 Artificial Cell Engineering Technology Research Center, Tianjin, China; 5 Tianjin Institute of Hepatobiliary Disease, Tianjin, China; 6 Department of Hepatology and Gastroenterology, Tianjin Union Medical Center Affiliated to Nankai University, 190, Jieyuan Road, Hongqiao District, Tianjin, China; 7 Fourth Department of Liver Disease (Difficult & Complicated Liver Diseases and Artificial Liver Center), Beijing You’an Hospital Affiliated to Capital Medical University, Beijing, China; 8 Beijing Municipal Key Laboratory of Liver Failure and Artificial Liver Treatment Research, Beijing, China; 9 The Fifth Medical Center of Chinese PLA General Hospital, Beijing, China

**Keywords:** Acute-on-chronic liver failure, Hepatitis B virus reactivation, Precipitating events, Prognosis

## Abstract

**Background:**

The manifestations and prognoses of acute-on-chronic liver failure (ACLF) with different precipitating events remain heterogeneous. We aimed to investigate the characteristics and prognosis of patients with hepatotropic viral insult (HVI)-induced hepatitis B-related ACLF (HBV-ACLF).

**Methods:**

452 patients with confirmed diagnosis of ACLF were screened in three medical centers in China, and 203 HBV-ACLF patients with definite acute precipitating events were retrospectively analyzed. According to the precipitating events, HBV-ACLF patients induced by HBV reactivation and super-infection with HAV were classified as the hepatotropic viral insult group and those induced by other factors, as the non-virus insult (NVI) group. The clinical characteristics, predictive scoring model, and prognosis of the two groups were compared.

**Results:**

Hepatitis B virus reactivation accounted for the largest proportion (39.9%) among all precipitating events. Exacerbation time frame of the HVI group was significantly longer than that of the NVI group (20 days vs. 10 days, P < 0.001). Comparison of intergroup prognosis showed that there was no significant difference in the 28 day mortality (20.9 vs. 13.7%, P = 0.125), while the 90 day and 1 year mortality in the HVI group were higher than those in the NVI group (36.3 vs. 24.4%, P = 0.014; 39.5% vs. 27.5%, P = 0.020, respectively). In the HVI group, the lactic acid-free APASL-ACLF Research Consortium (AARC) had better predictive value for 90 day mortality (0.741).

**Conclusions:**

The 90 day and 1 year survival rate was lower in HBV-ACLF patients induced by HVI than by NVI. The lactate-free AARC score was a better predictor of short- and long-term prognosis in patients with HVI-induced HBV-ACLF.

## Background

Acute-on-chronic liver failure (ACLF) is a complex clinical syndrome with acute deterioration of liver function, organ failure, and high short-term mortality, which is considered distinct from acute decompensation of cirrhosis [1, 2]. At present, because of the differences in the etiology of liver disease in Europe, USA, and the Asia Pacific region, there is no consensus yet on the diagnostic criteria of ACLF [3]. Given that patients with ACLF represent a heterogeneous population, previous studies focused on the clinical characteristics of ACLF patients with different precipitating events. However, the conclusions were inconsistent. Shi et al. reported that the 90 day and 1 year survival time of ACLF caused by extrahepatic insults were lower than that caused by hepatic injury [4]. However, Yin et al. suggested that there was no difference in short-term mortality of patients with ACLF between hepatic and extrahepatic precipitating events [5]. Fernandez et al. suggested that the risk of aggravation in patients with bacterial infection-induced ACLF was significantly higher than that of patients without bacterial infection, and the 90 day accumulative survival of the former was significantly lower than the latter [6]. It should be noted that all the above studies were aimed at ACLF populations based on different criteria, wherein the etiology of ACLF is not specifically defined. In China, chronic hepatitis B is the main cause of ACLF [7]. Hepatotropic viral infection includes hepatitis B virus (HBV) reactivation and super-infection with hepatitis A virus (HAV) or hepatitis E virus (HEV) and is the major cause of acute insults for precipitating ACLF [8]. Studies on the characteristics and prognosis of the HBV-ACLF patients induced by hepatotropic viral infection are limited. Therefore, we aimed to analyze the characteristics and prognosis of HBV-ACLF patients with definite hepatotropic viral infection from multiple centers in China, according to the enrolment standards of the Asian Pacific Association for the Study of Liver (APASL) consortium.

## Methods

### Study population

Patients who fulfilled the APASL ACLF criteria from a retrospective cohort referred between December 2012 and February 2019 in The Third Central Hospital of Tianjin, Beijing You'an Hospital Affiliated to Capital Medical University, and The Fifth Medical Center of Chinese PLA General Hospital were screened. According to APASL recommendations [8], 452 patients who were diagnosed with ACLF failure, defined as acute severe hepatic damage (total bilirubin [TBIL] ≥ 5 mg/dL and international normalized ratio [INR] ≥ 1.5 or prothrombin activity [PTA] ≤ 40%) accompanied by ascites and/or hepatic encephalopathy [HE] within 4 weeks with previously diagnosed chronic liver disease or cirrhosis, were screened. The exclusion criteria were as follows: (1) age < 18 years; (2) non-HBV related liver disease; (3) combined with hepatocellular carcinoma (HCC) or extrahepatic malignancies; (4) combined with hepatitis C virus (HCV) or human immunodeficiency virus (HIV) infection; (5) patients with no definite precipitating event; (6) those who had received a liver transplant; and (7) those whose hospital stay was < 1 day.

The diagnostic criteria of chronic hepatitis B refers to the Guideline of Prevention and Treatment for Chronic Hepatitis B of China [9]: positive for the HBV surface antigen ≥ 6 months. Cirrhosis was diagnosed based on previous liver biopsy results, clinical evidence of previous laboratory tests, and endoscopic (esophageal and gastric varices) and radiological imaging of portal hypertension. Overt ascites was diagnosed by ultrasonography. Hepatic encephalopathy was defined and graded based on the West Haven criteria [10]. Exacerbation time refers to the time from the precipitating event to the occurrence of liver failure. The type of ACLF were divided into A, B, and C according to the basic liver disease as non-cirrhosis, compensated cirrhosis or decompensated cirrhosis, respectively [11].

### Data collection

We collected the following clinical and demographic information in a retrospective database datasheet: age, sex, history, exacerbation time, precipitating events, laboratory parameters, and mortality at 28 days, 90 days, and 1 year. For all patients, laboratory data were collected at days 1, 7, 14, and 28 after diagnosis of ACLF.

### Definition of precipitating events and grouping

The enrolled patients had a record of definite precipitating events (PEs). The eight potential PEs were defined as follows:

1. HBV reactivation is defined as an increase in HBV DNA level by ≥ 2 log IU/mL from baseline or reappearance of HBV DNA viremia or HBsAg in those with previously detectable HBV DNA or HBsAg owing to withdrawal of antiviral drugs and resistance to nucleotide analogues or after treatment of immunosuppressive and chemotherapeutic drugs [9, 12];
2. Super-infection with HAV and HEV was diagnosed by the presence anti-HAV/HEV serum immunoglobulin IgM and IgG [13];
3. Hepatotoxic drugs’ insult was defined as the use of hepatotoxic drugs such as antituberculotic drugs, non-steroidal anti-inflammatory drugs, or herbal medicine within 3 months before diagnosis of ACLF, and a strong temporal relationship between exposure to the drug and development of ACLF [14];
4. Active alcohol consumption was defined as having more than 14 drinks per week in women and more than 21 drinks per week in men, within 6 months prior to admission [2];
5. Upper gastrointestinal bleeding (UGIB) was defined as active bleeding due to esophageal and gastric varices within 1 month before diagnosis of ACLF.
6. Bacterial infection was defined as previously described [15] including spontaneous peritonitis, intestinal infections, lung infections, urinary tract infections, or bacteremia, when they were detected prior or at the time of diagnosis of ACLF;
7. Overwork was defined as a patient's self-reported history of moderate intensity physical and mental work beyond the normal working hours within 4 weeks before the diagnosis of ACLF [16, 17].
8. Surgery was defined as the surgical operation under general anesthesia.

Acute precipitating events were categorized into two groups. HBV reactivation and super-infection with HAV or HEV were classified as the hepatotropic viral insult (HVI) group and the other factors as the control group or non-virus insult (NVI) group.

### Calculation of liver scoring systems

The following five prognostic models were used to analyze the prognosis of ACLF patients with different PEs included: Child–Pugh score (CTP: range, 0–15) [18]; Model for End-Stage Liver Disease (MELD: range, 6–40) [19]; Chronic Liver Failure-Sequential Organ Failure Assessment (CLIF-SOFA); CLIF-Consortium-ACLF (CLIF-ACLF); and APASL-ACLF Research Consortium (AARC). CLIF-SOFA score (range, 0–24) consists of six organ systems and is proposed to evaluate organ failures in ACLF patients [2]. CLIF-ACLF score is adjusted based on the CLIF-SOFA scale and calculated as follows: 10 × [0.33 × CLIF-OFs + 0.04 × Age + 0.63 × Ln(WBC count)–2] [20]. AARC-ACLF score is recommended by APASL as a reliable prediction model for ACLF patients, which is measured by HE, serum bilirubin, INR, lactate, and creatinine [21]. However, in this study, we used lactate-free AARC instead because the serum lactate was not a routine test parameter in some areas.

### Statistical analysis

Continuous variables were expressed as mean ± standard deviation (SD) or median with interquartile range (IQR). Continuous data between two groups were compared by Student’s *t*-test or Mann–Whitney U test. Comparison of categorical variables was carried out by chi-square test. The 28 day, 90 day, and 1 year mortality curves of different groups were analyzed using the log-rank test. Predictors for the 28 day and 90 day mortality were analyzed by univariate and multivariate Cox regression model. Dynamic changes of continuous variables between groups were compared using repeated measurements. ROC curve was used to explore the prediction model. Statistical analyses were performed using SPSS (23.0; SPSS, Inc., Chicago, IL). P < 0.05 was considered to indicate statistical significance.

## Results

### Clinical characteristics

**Fig. 1:**
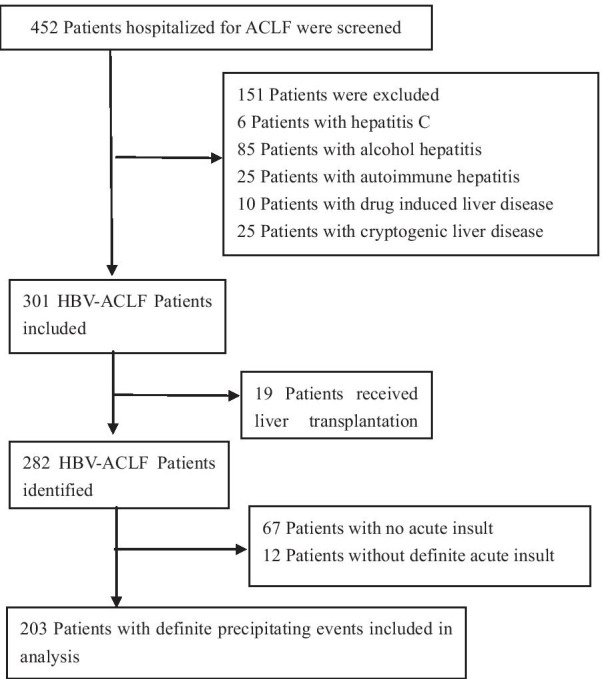
Flowchart of enrollment of HBV-related ACLF cohort

Of 452 patients with ACLF, 203 patients with HBV-ACLF with definite PEs were finally included (Fig. 1). Among the included patients, 186 were identified at admission, and 17 patients with cirrhosis developed ACLF within 14 days after admission. The mean age of the patients was 47.7 years. Jaundice and fatigue were the main symptoms of all patients. The mean time of disease deterioration was 14 days. The 28 day, 90 day, and 1 year mortality of all ACLF patients were 18.2, 32, and 35.5%, respectively.

Types of PEs among all patients are listed in Table 1. HBV reactivation accounted for the largest proportion (39.9%) in all precipitating events. One patient with hepatitis A virus superinfection was classified into the hepatotropic viral insult group, while no patients with hepatitis E, hepatitis C or hepatitis D virus superinfection were found in our cohort.

**Table 1:** Etiology of precipitating events

Precipitating events	Frequency
Hepatotropic viral insult(N = 82)	
HBV reactivation, no (%)	81 (39.9)
Withdrawal of antiviral drugs	57 (28.1)
Due to immunosuppressive/chemotherapeutic drugs (5.9%), or viral mutations, chemotherapeutic drugs	12 (5.9)
Viral mutations	12 (5.9)
Super-infection with HAV, no (%)	1 (0.5)
Non-virus insult (N = 121)	
Bacterial infection, no (%)	60 (29.6)
Drug insult, no (%)	21 (10.3)
Active alcohol drinking, no (%)	21 (10.3)
Overwork, no (%)	16 (7.9)
Upper gastrointestinal bleeding, no (%)	2 (1.0)
Surgery, no (%)	1 (0.5)

*HBV* hepatitis B virus, *HAV* hepatitis A virus

In 81 patients with HBV reactivation,57 (28.1%) patients had hepatitis B virus flare due to discontinuation of antiviral drugs, with an average withdrawal time of 24 weeks. 12 (5.9%) patients had virological breakthrough during the use of antiviral drugs and detected viral mutations. All these patients switched or added nucleoside analogs according to the instructions of viral mutation detection, (entecavir alone 0.5 mg, tenofovir alone 300 mg, telbivudine 600 mg/lamivudine 100 mg/ entecavir 0.5 mg plus adefovir 10 mg or tenofovir 300 mg daily)0.12 (5.9%) patients with HBV reactivation due to the application of immunosuppressive drugs or chemotherapy drugs were immediately started antiviral treatment (entecavir 0.5 mg or tenofovir 300 mg daily). 85% of surviving patients achieved more than 2 logs reduction of HBV DNA levels at day 28 after antiviral treatment.

Among the NVI group, the proportion of acute exacerbation caused by bacterial infection was the highest (29.6%), followed by drug insult (10.3%), active alcohol drinking (10.3%), and overwork (7.9%). In addition, one patient (0.5%) underwent surgical operation.

### Comparison of clinical characteristics between the HVI and NVI groups

Comparison of the clinical characteristics between the HVI and NVI group are shown in Table 2. There were no significant intergroup differences in age, sex, and history of cirrhosis. Exacerbation time frame in the HVI group was significantly longer than that in the NVI group (20 days vs. 10 days, P < 0.001). At the time of ACLF diagnosis, the level of albumin in the HVI group was significantly higher than that in the NVI group, and there was no significant difference in other parameters of hepatic and renal function. There were no significant intergroup differences in the incidence of organ failure, baseline Child–Pugh, MELD, CLIF-SOFA, CLIF-ACLF, and lactate-free AARC scores.

**Table 2:** Comparison of clinical features and prognosis between hepatotropic viral insult and non-virus insult groups

	Total (n = 203)	HVI (n = 82)	NVI (n = 121)	P value
Age,years	47.7 ± 11.4	49.3 ± 9.6	46.6 ± 12.4	0.087
Male,no(%)	161(79.3)	64(73.1)	97(80.2)	0.715
ACLF Type, no (%) A B C	59 (29.1) 75 (36.9) 69 (34.0)	24 (29.3) 34 (41.5) 24 (29.3)	35 (28.9) 41 (33.9) 45 (37.2)	0.435
Exacerbation time frame, days, median (IQR)	14 (23)	20 (20)	10 (14)	0.003
Laboratory data				
ALT, IU/L median (IQR)	185.0 (470.5)	151.7 (535.7)	219 (472.5)	0.927
AST, IU/L median (IQR)	203.3 (374.4)	184.5 (460.4)	207.0 (394.1)	0.785
Albumin, g/L mean(SD)	29.9 ± 5.1	31.5 ± 4.8	28.9 ± 5.1	0.000
GGT,IU/L median (IQR)	85.0 (83.8)	92.0 (101)	82.0 (77.5)	0.091
TB, umol/L median (IQR)	263.3 (224.4)	285.8 (215.4)	252.4 (216.6)	0.216
Na, mmol/L median (IQR)	135.1 (5.5)	134.6 (5.5)	135.2 (5.8)	0.788
Creatinine, umol/L median (IQR)	72.0 (37.0)	68 (36.5)	76(38.5)	0.195
WBC, 10^9^/L median (IQR)	6.2 (4.5)	5.8 (8.6)	6.8 (4.2)	0.227
NEUT, % median (IQR)	72.0 (14.6)	71.4 (15.3)	72.0 (15.0)	0.880
Platelet, 109/L median (IQR)	79 (70)	79 (117)	80 (61)	0.845
PTA,% median (IQR)	33.8 (13.6)	33.6(14.3)	34 (13.2)	0.896
INR median (IQR)	2.1 (0.8)	2.1 (0.9)	2.1 (0.8)	0.707
Severity score				
CTP median (IQR)	11.0( 2)	11.0 (2)	11.0 (2)	0.134
MELD mean (SD)	23.6 ± 5.4	23.7 ± 6.1	23.5 ± 4.9	0.830
CLIF-SOFA median (IQR)	7 (1)	7 (2)	7 (1)	0.660
Lactate free AARC median (IQR)	7 (1)	9 (2)	8 (3)	0.738
CLIF-ACLF median (IQR)	39.2 (12.7)	41.3 (11.7)	38.3 (12.8)	0.188
Cumulative mortality				
28 day, no (%)	37 (18.2)	19 (20.9)	18 (13.7)	0.125
90 day, no (%)	65 (32.0)	33 (36.3)	32 (24.4)	0.020
1 year, no (%)	72 (35.5)	36 (39.5)	36 (27.5)	0.014

*HVI* hepatotropic viral insult, *NVI* non-virus insult, *ACLF* acute-on-chronic liver failure, *ALT* alanine aminotransferase, *AST* aspartate aminotransferase, *GGT* gamma glutamyl transferase, *TB* total bilirubin, *Na* serum sodium, *WBC* white blood cell, *NEUT* neutrophils, *PTA* prothrombin activity, *INR* international normalized ratio, *CTP* child-turcotte-pugh, *MELD* model for end-stage liver disease, *CLIF-SOFA* chronic liver failure-sequential organ failure assessment. *AARC* APASL-ACLF research consortium, *CLIF-ACLF* chronic liver failure -consortium- acute-on-chronic liver failure score

### Comparison of prognosis and mortality between hepatotropic viral insult group and non-virus insult group

**Fig. 2:**
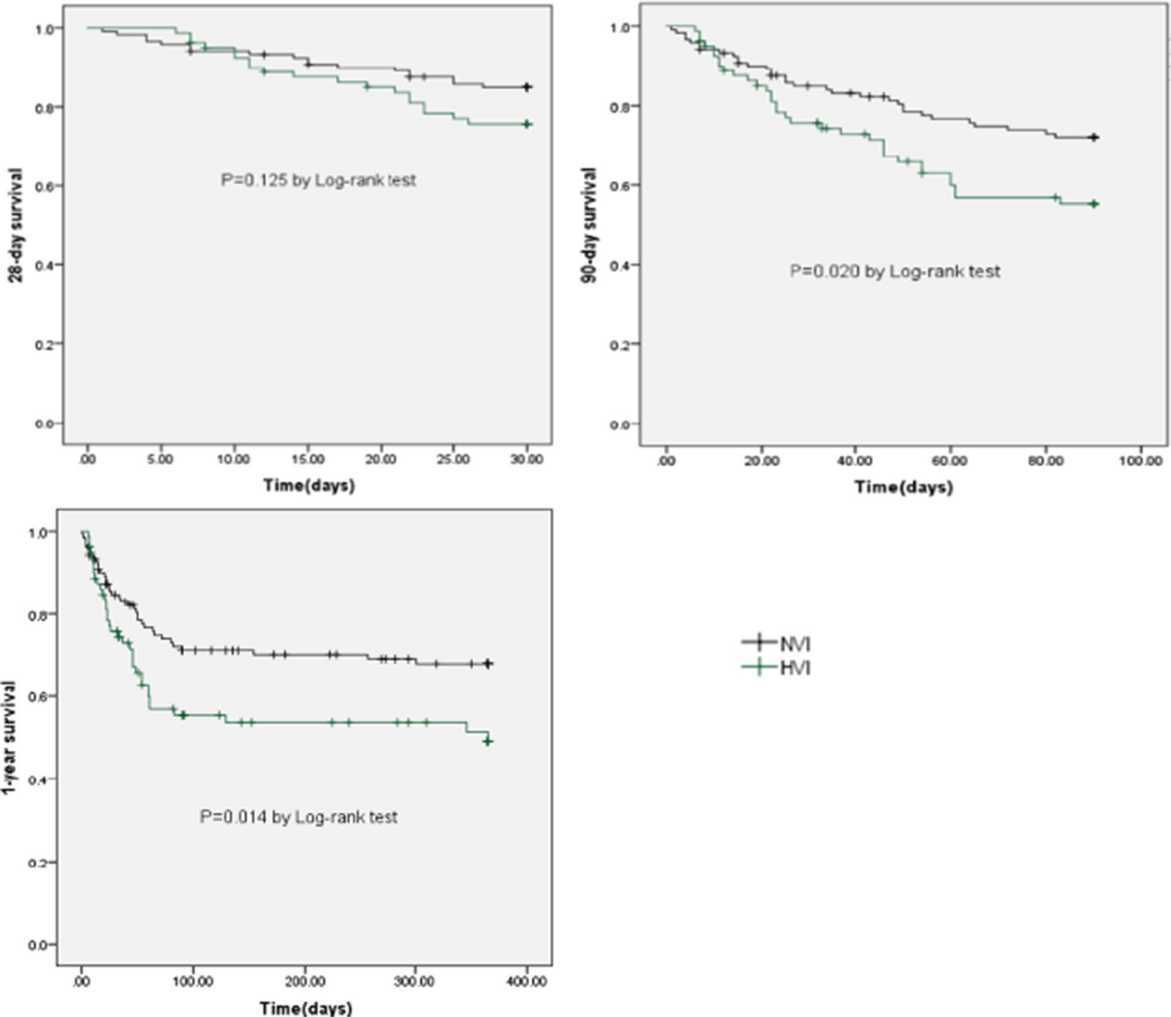
Comparison of the 28 day, 90 day and 1 year survival between hepatotropic viral insult group and non-virus insult group. *HVI* hepatotropic viral insult, *NVI* non-virus insult

**Fig. 3:**
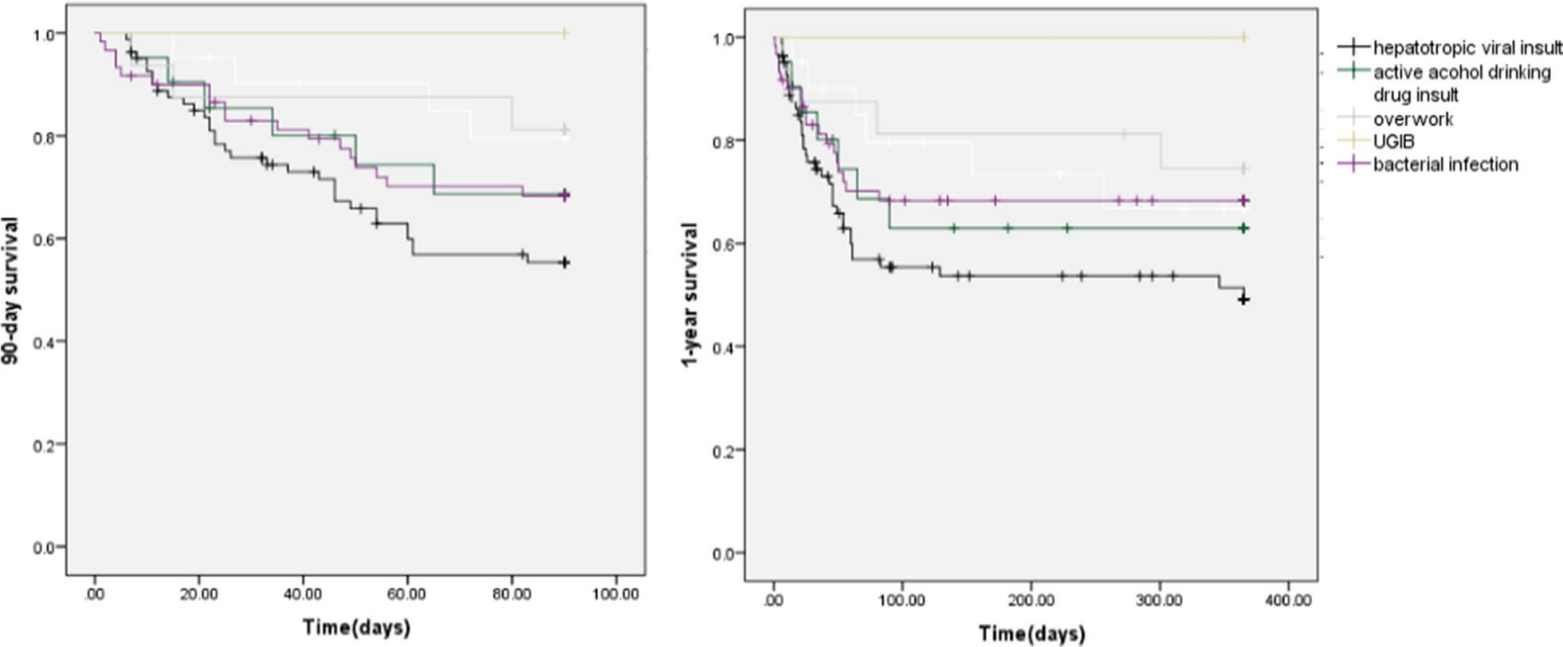
Comparison of the 90 day and 1 year survival of patients induced by hepatotropic viral insult and active alcohol drinking, hepatotoxic drugs, overwork, UGIB and bacterial infection group. *UGIB* upper gastrointestinal bleeding

There was no difference in the 28 day mortality (20.9% vs. 13.7%, P = 0.125) between the two groups. However, the 90 day and 1 year mortality were significantly higher in the HVI group than the NVI group (36.3% vs. 24.4%, P = 0.020; 39.5% vs. 27.5%, P = 0.014; respectively) (Table 2, Fig. 2). The 90 day and 1 year survival of patients with HVI-induced ACLF were lower than those induced by other insults (Fig. 3).

Predictors of 28 and 90 day mortality in the two groups were analyzed by multivariate cox regression. ACLF Type, bilirubin, and PTA were independent predictors of 28 day mortality, while age, PTA, and creatinine (Cr) were independent predictors for 90 day mortality in the HVI group. (Table 3).

**Table 3:** Risk factors associated with 28 day and 90 day mortality by a Multivariate COX’s Hazard Model in HVI and NVI groups

	28 day mortality			90 day mortality		
Variables	Hazard ratio	95%CI	P Value	Hazard ratio	95%CI	P value
HVI (n = 82)						
ACLF Type	0.231	0.061–0.870	0.030	1.283	0.722–2.279	0.395
Age	1.053	0.989–1.122	0.107	1.073	1.024–1.125	0.003
TB	1.004	1.001–1.007	0.020	1.002	0.999–1.004	0.129
PTA	0.934	0.878–0.993	0.029	0.943	0.900–0.988	0.013
Creatinine	1.002	0.998–1.007	0.349	1.005	1.002–1.008	0.003
Na	1.049	0.951–1.157	0.335	0.977	0.920–1.138	0.457
WBC	0.781	0.887–1.173	0.781	1.000	0.889–1.125	0.999
Neut	1.003	0.951–1.059	0.903	0.986	0.948–1.126	0.497
NVI (n = 121)						
ACLF Type	0.506	0.142–1.802	0.293	0.386	0.134–1.113	0.078
Age	1.028	0.985–1.073	0.208	1.014	0.980–1.050	0.426
TB	1.003	0.999–1.006	0.110	1.003	1.000–1.005	0.053
PTA	0.952	0.897–1.010	0.100	0.968	0.928–1.010	0.128
Creatinine	1.007	0.992–1.021	0.368	1.011	0.999–1.023	0.062
Na	1.016	0.929–1.021	0.722	1.009	0.943–1.079	0.804
WBC	1.010	0.935–1.090	0.802	0.996	0.934–1.062	0.904
Neut	1.034	0.977–1.094	0.249	1.039	0.996–1.084	0.074

*HVI* hepatotropic viral insult, *NVI* non-virus insult, *TB* total bilirubin, *PTA* prothrombin activity, *Na* serum sodium, *WBC* white blood cell, *NEUT* neutrophils

**Fig. 4:**
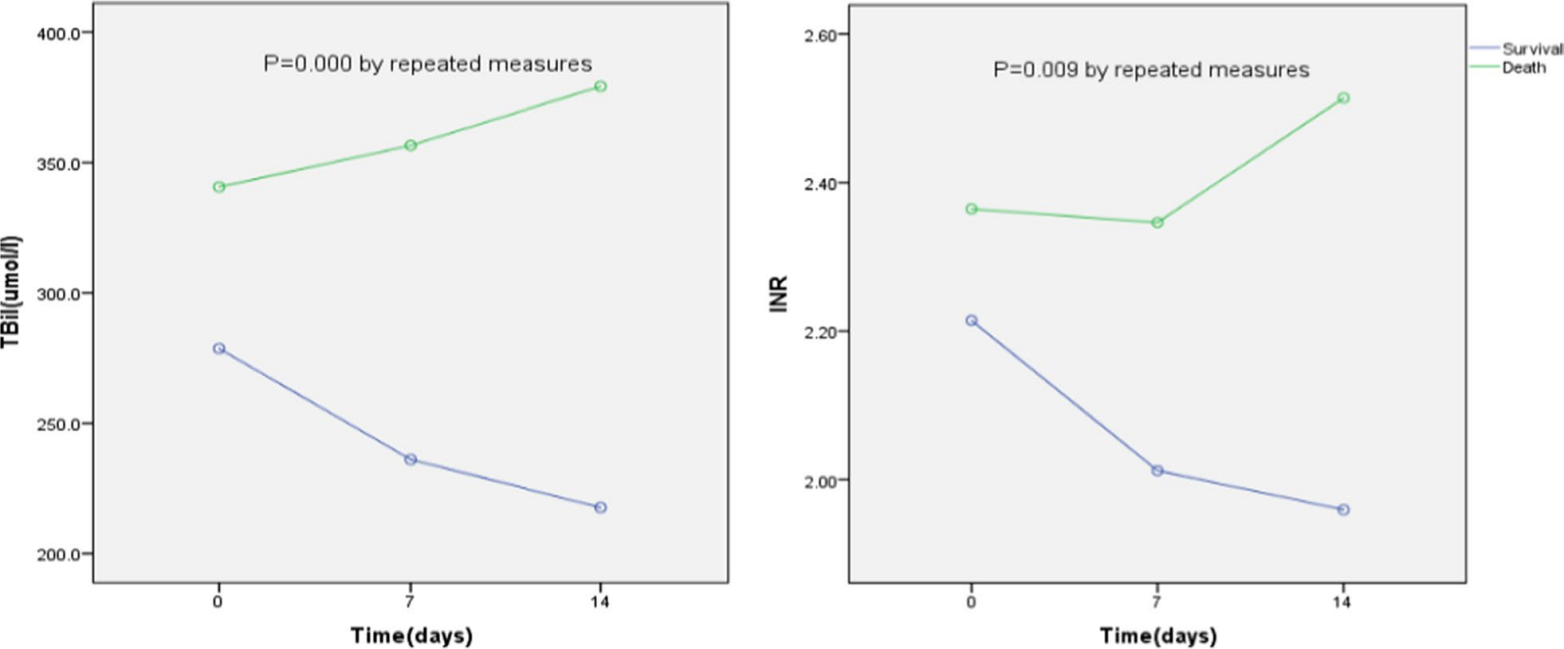
Dynamic changes of TBil and INR within 14 days after ACLF diagnosis of NVI group

The clinical parameters of the 1st, 7th, and 14th day after ACLF diagnosis were measured dynamically and compared between patients who died or survived at 90 days in both groups. TBil, INR, white blood cell count (WBC), and serum sodium (Na) and creatinine levels showed significant dynamic changes between the deceased and survived patients in the NVI group within 2 weeks after admission. However, these parameters were not significantly different in the HVI group (Table 4 and Fig. 4).

**Table 4:** Dynamic changes of parameters within 14 days after ACLF diagnosis in HVI and NVI groups

**Variables**	F	P value
HVI (n = 82)		
TB	2.98	0.090
PTA	5.876	0.019
INR	2.763	0.102
Creatinine	2.697	0.107
WBC	0.296	0.589
Na	0.618	0.435
NVI (n = 121)		
TB	13.544	0.000
PTA	4.417	0.039
INR	7.051	0.009
Creatinine	6.633	0.012
WBC	1.250	0.267
Na	6.098	0.016

*HVI* hepatotropic viral insult, *NVI* non-virus insult, *TB* total bilirubin, *PTA* prothrombin activity, *INR* international normalized ratio, *WBC* white blood cell, *NEUT* neutrophils

### Comparison of prognostic models between the HVI and NVI groups

**Fig. 5:**
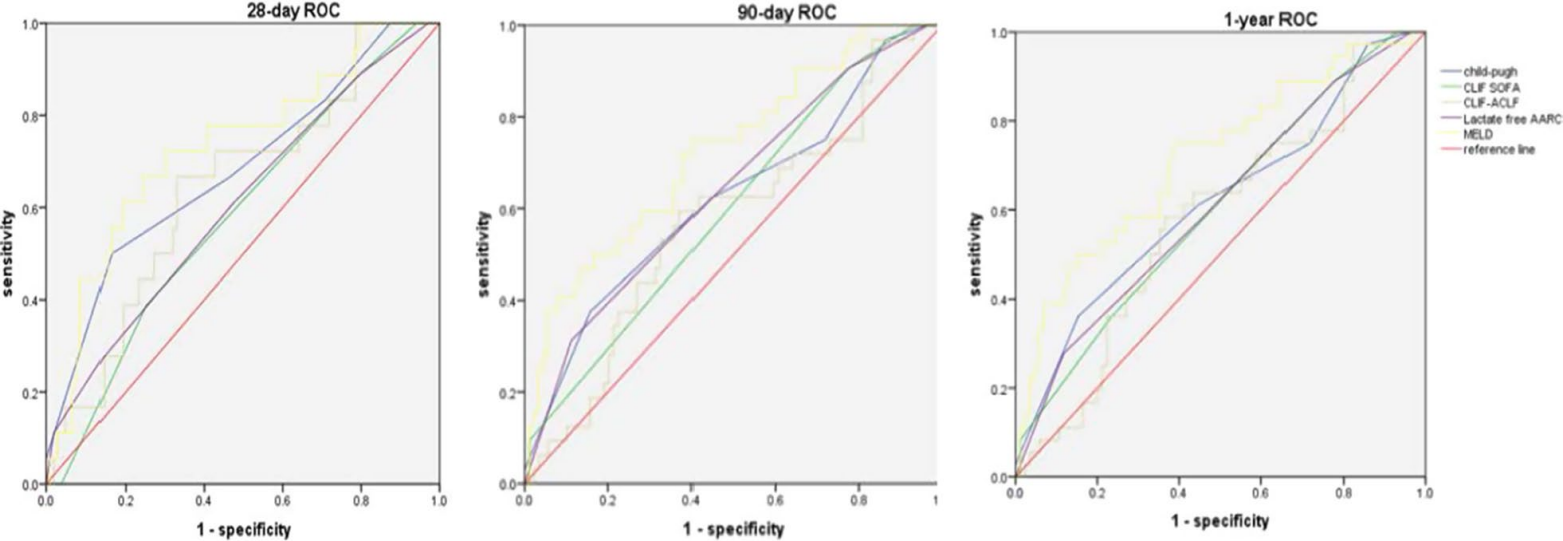
ROC curve of prognostic models in 28 day, 90 day and 1 year mortality in NVI group

**Fig. 6:**
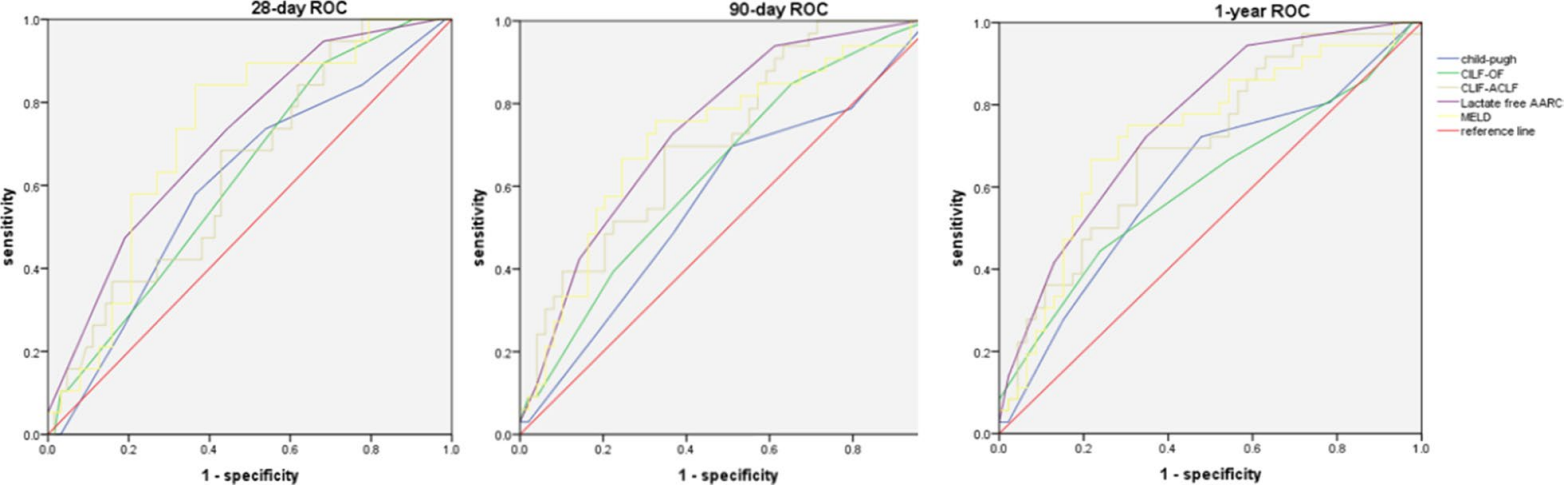
ROC curve of prognostic models in 28 day, 90 day and 1 year mortality in HVI group. *ACLF* acute-on-chronic liver failure, *CTP* child-turcotte-pugh, *MELD* model for end-stage liver disease, *CLIF-SOFA* chronic liver failure-sequential organ failure assessment, *AARC* APASL-ACLF research consortium, *CLIF-ACLF* chronic liver failure -consortium- acute-on-chronic liver failure score

Five prognostic model scores were used to assess the 28 day, 90 day, and 1 year mortality prediction of ACLF patients in the two groups. In the NVI group, the MELD model had the highest receiver operating characteristic (ROC) curve (28 day: 0.730, 90 day: 0.722, 1 year: 0.714), and the ROC in other four scores were lower than 0.7. Furthermore, for patients in the HVI group, lactate-free AARC had the highest ROC (90 day: 0.741, 1 year: 0.758), followed by MELD (28 day: 0.723, 90 day: 0.719, 1 year: 0.726), and CLIF-ACLF (90 day: 0.712, 1 year: 0.700) (Table 5, Figs. 5 and 6).

**Table 5:** Comparison of prediction of five scoring models on 28 day, 90 day, and 1 year mortality in two groups

	28 day mortality		90 day mortality		1 year mortality	
Variables	OR(95%CI)	P value	OR(95%CI)	P value	OR(95%CI)	P value
NVI (n = 121)						
CTP	0.681 (0.539–0.823)	0.015	0.622 (0.504–0.740)	0.041	0.618 (0.505–0.731)	0.040
CILF-SOFA	0.585 (0.448–0.721)	0.253	0.599 (0.487–0.711)	0.097	0.603 (0.494–0.712)	0.074
CLIF-ACLF	0.644 (0.509–0.779)	0.052	0.573 (0.457–0.689)	0.222	0.579 (0.469–0.688)	0.172
Lactate free AARC	0.607 (0.463–0.751)	0.149	0.641 (0.528–0.753)	0.018	0.610 (0.500–0.720)	0.056
MELD	0.730 (0.600–0.860)	0.002	0.722 (0.619–0.826)	0.000	0.714 (0.611–0.817)	0.000
HVI (n = 82)						
CTP	0.603 (0.460–0.746)	0.175	0.571 (0.443–0.699)	0.279	0.617 (0.493–0.741)	0.071
CILF-SOFA	0.622 (0.488–0.755)	0.110	0.637 (0.516–0.758)	0.036	0.640 (0.521–0.760)	0.030
CLIF-ACLF	0.638 (0.504–0.772)	0.070	0.712 (0.601–0.823)	0.001	0.700 (0.586–0.813)	0.002
Lactate free AARC	0.714 (0.588–0.740)	0.005	0.741 (0.635–0.848)	0.000	0.758 (0.655–0.861)	0.000
MELD	0.723 (0.602–0.843)	0.003	0.719 (0.603–0.834)	0.001	0.726 (0.6154–0.839)	0.000

*HVI* hepatotropic viral insult, *NVI* non-virus insult, *ACLF* acute-on-chronic liver failure, *CTP* child-turcotte-pugh, *MELD* model for end-stage liver disease, *CLIF-SOFA* chronic liver failure-sequential organ failure assessment, *AARC* APASL-ACLF research consortium, *CLIF-ACLF* chronic liver failure -consortium- acute-on-chronic liver failure score

## Discussion

ACLF is a clinical syndrome characterized by acute deterioration of liver function based on chronic liver disease which is associated with poor outcome in response to an acute insult [1]. At present, there is no consistent definition of ACLF owing to the different etiology of chronic liver disease between the Asian and European populations [22]. Chronic hepatitis B is the main cause of chronic liver disease in Asia, especially in China, while alcoholic liver disease is the most common cause in Europe and USA [23, 24]. In addition, the prognosis of ACLF with various PEs and different underlying chronic diseases is not unanimous. Therefore, it seemed necessary to assess the prognosis of different acute insults in a homogeneous ACLF population. The HBV-ACLF patients with definite PEs included in this study were enrolled according to the APASL criteria of ACLF, from three medical centers of China. For patients with HBV-ACLF, acute liver deterioration induced by hepatitis B virus exacerbation was still the predominant factor [25] and the mechanism of liver injury was different from other factors, which should be analyzed separately.

There have been previous studies on the PEs of ACLF, in which PEs were divided into hepatic-ACLF and extrahepatic-ACLF groups [4]. The former was induced by hepatic insults such as acute exacerbation or flare-up of chronic hepatitis B(CHB), superimposed infection of HAV or HEV, active drinking of alcohol, and use of hepatotoxic drugs, whereas the extra hepatotropic insults were considered as bacterial infection, UGIB, and other surgical factors. However, it was difficult to simply divide the PEs into hepatic and extrahepatic insults, because the extent of alcohol consumption and duration of active drinking to be included as acute insult was unclear. With prolonged drinking time, autoimmune injury will gradually manifest besides direct liver injury [8, 26]. The proportion of drug insult was 10.3% in our study, which was consistent with a previous report of the AARC database [8]. Although drugs have been listed as a precipitating factor in ACLF, different types of drugs have heterogeneous effects on liver injury. In this study, bacterial infection as a PE also accounted for a higher proportion, whereas various bacterial and fungal infections may directly affect the liver in addition to spreading from extrahepatic organs [27]. It remains to be discussed whether hemorrhage or manifestation of liver failure is the PE [28, 29]. In our study, both cases of hemorrhagic events occurred before liver function damage, which may be related to acute hepatic ischemia and subsequent bacterial infection. Overwork was included as a precipitating event in this study, which was not mentioned in previous studies. 7.9% of all patients had a definite history of overwork in the last month before admission, as shown by a retrospective investigation, wherein overwork was manifested as physical work in excess of normal time and intensity, mental stress, and sleep disorders and links to cardiovascular and cerebrovascular illnesses and deaths [30, 31]. We assume that overwork may also be a cause for disease progression and the likely potential for liver injury related to psychological stress and changes in the internal environment [32], however further work is needed to confirm the impact of overwork as a PE on ACLF. The effects of these heterogeneous factors on liver deterioration in patients with underling hepatitis B or cirrhosis were varied, but they were distinguished from hepatitis B virus reactivation. Therefore, we classified them as NVIs.

ACLF cases in the HVI group were mainly related to HBV reactivation. We found that exacerbation time in the HVI group was significantly longer than in the NVI group. It took longer for liver deterioration induced by reactivation of HBV in the HVI group than in that NVI group. Jaundice and coagulopathy, which define liver failure, were the main manifestations of organ failure, and the proportion of extrahepatic organ failure was similar in the two groups.

The 90 day and 1 year survival in the HVI group was lower than that in NVI group; there was no difference in the 28 day mortality. The results of this study are inconsistent with those published by Shi et al., which may likely be due to the different ACLF diagnostic criteria and study population. Our study only focused on the HBV-ACLF patients with the ACLF criteria of APASL. The 90 day and long prognosis of liver failure induced by HVI were worse in this study. For HBV-ACLF, effective control of HBV replication makes sense for the recovery of liver injury induced by HBV reactivation [33]. Early and rapid reduction of HBV titer is the essence of therapy, which is related to the suppression of hepatocyte inflammatory necrosis [34]. However, it is suggested that for HBV-ACLF patients, the basic liver function at the start of antiviral treatment is more important for prognosis than the antiviral regimens. Some studies suggested that antiviral therapy was beneficial to ACLF patients with MELD scores of 20–30 [35]. Therefore, baseline liver status may have a positive impact on the prognosis of ACLF patients with HBV reactivation. In this study, Type B, bilirubin, PTA, age, and serum creatinine at baseline were independent predictors for the 28- and 90 day mortality.

Patients with non-virus precipitants such as bacterial infection, alcohol consumption, hepatotoxic drugs, and bleeding could benefit from elimination of these causative factors, by considering antibiotic therapy [6], quitting drinking, taking drugs for interruption of liver damage, or undergoing hemostasis therapy. Therefore, the short-term therapy may have an impact on the survival and death of patients. A previous study reported that the patient’s condition during the first 3–7 days after ACLF diagnosis determined their prognosis [36]; therefore, we focused on the parameter changes within one week after diagnosis due to their positive predictive prognosis. In this study, there were significant dynamic changes in bilirubin, PTA, INR, creatinine, and blood sodium within 14 days between the patients who survived and those that died in the NVI group, but there was no significance seen in the HVI group. We speculated that the dynamic changes of liver and renal function in the NVI group within 14 days after admission might be related to the prognosis.

We attempted to compare the predictive scoring model for HBV-ACLF patients induced by hepatotropic virus insult. The MELD score, which is used to assess end-stage liver disease, had good predictive value both for short- and long-term mortality in the two groups. The results revealed that MELD score was superior to the other five models in predicting the prognosis of patients in the NVI group. Lactate-free AARC score showed higher predictive value than for the existing models for the 28 day, 90 day, and 1 year mortality in the NVI group. The AARC score, which included TBil, INR, grade of HE, plasma lactate, and serum creatinine, could reliably predict the severity and outcome for ACLF patients. However, serum lactate test is not a routine test in some countries. A study on the predictive value of lactate-free AARC score among 749 patients with alcoholic liver disease and liver failure (from the AARC database) [37] suggested that the AUC prediction values for 90 day mortality is similar to that of other models. The lactate-free AARC score could also reflect liver failure to some extent, which is at the core of ACLF, based on APASL criteria. In the HVI group, we found that compared with MELD and CLIF-ACLF, the lactate free AARC score was a better predictor of 28 day, 90 day, and 1 year mortality. However, the AUROC was less than 0.80, therefore we still need to find more ideal biomarkers and models for prognosis prediction.

There are some limitations in this study. First, although patients were enrolled from three Chinese hospitals, the sample size was still relatively small. In this study, part of the screened HBV-ACLF patients lacked the PE, and some patients had ambiguous insults; both these categories of patients were excluded. Consequently, there are some deviations in the results of this study. Second, owing to the lack of lactate data, we scored both groups using lactate-free AARC. Therefore, it is necessary to expand the sample size to validate the results reported.

## Conclusions

In summary, HBV reactivation is still the most common PE in HBV-ACLF patients, and the 90 day and 1 year prognosis of patients with HVI-induced HBV-ACLF was worse than that induced by non-virus insult. It is necessary to pay attention to the liver damage caused by HBV reactivation during the management of patients with chronic HBV infection. Lactate-free AARC score and MELD scores are valuable predictors of short- and long-term prognosis in patients with HVI-induced HBV-ACLF.

## Data Availability

The data that support the findings of this study are available from the corresponding author upon reasonable request.

## Abbreviations

ACLF: Acute-on-chronic liver failure
AARC: APASL-ACLF Research Consortium
APASL: Asian Pacific Association for the Study of Liver
NVI: Non-virus insult
HVI: Hepatotropic viral insult
HBV-ACLF: Hepatitis B-related acute-on-chronic liver failure
HAV: Hepatitis A virus
HBV: Hepatitis B virus
HCV: Hepatitis C virus
HEV: Hepatitis E virus
HCC: Hepatocellular carcinoma
CHB: Chronic hepatitis B
INR: International normalized ratio
PTA: Prothrombin activity
HE: Hepatic encephalopathy
HIV: Human immunodeficiency virus
PE: Precipitating event
UGIB: Upper gastrointestinal bleeding
WBC: White blood cell
ALT: Alanine aminotransferase
AST: Aspartate aminotransferase
GGT: Gamma glutamyl transferase
TB: Total bilirubin
Na: Serum sodium
NEUT: Neutrophils
PTA: Prothrombin activity
CTP: Child-turcotte-pugh
MELD: Model for end-stage liver disease;
CLIF-SOFA: Chronic liver failure-sequential organ failure assessment
CLIF-ACLF: Chronic liver failure -consortium- acute-on-chronic liver failure score
AARC: APASL-ACLF Research Consortium
AUROC: Area under the ROC curve
